# Behavioral aging is associated with reduced sensory neuron excitability in *Aplysia californica*

**DOI:** 10.3389/fnagi.2014.00084

**Published:** 2014-05-09

**Authors:** Andrew T. Kempsell, Lynne A. Fieber

**Affiliations:** Division of Marine Biology and Fisheries, Rosenstiel School of Marine and Atmospheric Science, University of MiamiMiami, FL, USA

**Keywords:** L-glutamate, NMDA, D-aspartate, pleural ganglion, buccal ganglion

## Abstract

Invertebrate models have advantages for understanding the basis of behavioral aging due to their simple nervous systems and short lifespans. The potential usefulness of *Aplysia californica* in aging research is apparent from its long history of neurobiological research, but it has been underexploited in this model use. Aging of simple reflexes at both single sensory neuron and neural circuit levels was studied to connect behavioral aging to neurophysiological aging. The tail withdrawal reflex (TWR), righting reflex, and biting response were measured throughout sexual maturity in 3 cohorts of hatchery-reared animals of known age. Reflex times increased and reflex amplitudes decreased significantly during aging. Aging in sensory neurons of animals with deficits in measures of the TWR and biting response resulted in significantly reduced excitability in old animals compared to their younger siblings. The threshold for firing increased while the number of action potentials in response to depolarizing current injection decreased during aging in sensory neurons, but not in tail motoneurons. Glutamate receptor-activated responses in sensory neurons also decreased with aging. In old tail motoneurons, the amplitude of evoked EPSPs following tail shock decreased, presumably due to reduced sensory neuron excitability during aging. The results were used to develop stages of aging relevant to both hatchery-reared and wild-caught Aplysia. Aplysia is a viable aging model in which the contributions of differential aging of components of neural circuits may be assessed.

## Introduction

Aging causes physiological changes in all major organ systems in the body. In the nervous system, human and animal model studies have shown altered neuronal physiology linked to changes in behavior. The study of neurobiological aging is complicated by the complexity of vertebrate nervous systems and lifespans not conducive to practical study design. Invertebrates such as *Caenorhabditis elegans, Drosophila melanogaster*, and *Aplysia californica* (Aplysia), therefore, have earned a place as relevant fundamental aging models owing to their simplified nervous systems, simple behaviors, and compact lifespans. An additional advantage of these models is the ability to measure behavioral and electrophysiological changes in defined neuronal circuits as a function of age.

In Aplysia, few studies have been published that correlate behavioral changes with altered nervous system functioning during aging. Deficits in the gill withdrawal reflex and osmoregulation with age have been linked to altered response properties in innervating motoneurons L7 and R15, including reduced input resistance and decreased responsiveness to synaptic input (Rattan and Peretz, 1981; Peretz, 1988). Recently, aging has been shown to cause specific changes in synaptic transmission in Aplysia, including reduced excitability and altered expression and composition of acetylcholine receptors during aging in the bursting neuron R15 of the abdominal ganglia (Akhmedov et al., 2013; Kadakkuzha et al., 2013). Components of the Aplysia nervous system have been shown to age at different rates. Cholinergic neurons R15, R2, and LPl1 of the abdominal ganglion have been found to have a unique subset of genes differentially expressed in older animals, with significant differences in gene expression between neuronal types during aging (Moroz and Kohn, 2010; Kadakkuzha et al., 2013). However, there has yet to be a study investigating the aging physiology of both sensory and motor neurons innervating behaviors in Aplysia. This study focused on aging of two reflexes, examining behavior, and sensory and motoneurons to gain insights about whether selective aging in one side of the circuit occurs, and, if so, whether it dominates aging in the reflex.

The two behaviors we studied were the tail-elicited tail withdrawal reflex (TWR) and the biting response. Tail withdrawal following a mechanical, chemical, or electrical stimulus is a monosynaptic reflex for which the ventral caudal cells of the pleural ganglion (PVC) are the primary mechanosensory neurons. These neurons make strong connections to ipsilateral pedal ganglion motoneurons innervating the tail (P5-7; Walters et al., 1983a). The PVC neuron arrangement within the pleural ganglion represents a crude somatotopic map of the ipsilateral surface of the body, and the mechanosensory neurons for the tail have been identified (Walters et al., 2004). These features make the TWR ideally suited for the study of aging-dependent changes at the behavioral and physiological levels.

Feeding behavior declines during aging, leading to the decrease in weight commonly seen in the weeks prior to death (Capo et al., 2003; Gerdes and Fieber, 2006; Fieber et al., 2010). Biting is controlled by identified mechanosensory neurons innervating the buccal mass, namely the left and right S clusters of the buccal ganglion (BSC), and dopaminergic motoneurons of the buccal ganglion (Kabotyanski et al., 1998), making the biting response another relevant model for correlations between behavioral and physiological aging.

Both these Aplysia sensory neuron types use glutamate (L-Glu) as their primary excitatory neurotransmitter (Dale and Kandel, 1993; Fox and Lloyd, 1999; Levenson et al., 2000; Ha et al., 2006). Applied L-Glu activated excitatory currents in PVC and BSC sensory neurons (Carlson and Fieber, 2011, 2012). D-Aspartate (D-Asp) is a relevant doppelganger of L-Glu (Olverman et al., 1988) whose physiological actions have recently been studied (Errico et al., 2008, 2011). In Aplysia, D-Asp activated a current pharmacologically distinct from ionotropic L-Glu receptor (iGluR) currents in PVC and BSC sensory neurons (Carlson et al., 2012). The frequency and density of D-Asp-induced currents decreased significantly in old PVC neurons (Fieber et al., 2010), suggesting that sensory neuron excitability is affected during aging in this model organism.

This study addressed whether simple reflexes in Aplysia age due to aging of specific components of the neural circuit before others to influence behavior. Since described sensorimotor synapses of Aplysia underlie well-studied reflex behaviors and are subject to synaptic facilitation and depression (Walters et al., 1983b; Baxter and Byrne, 2006), how these neuronal components age has wide relevance to Aplysia neurobiology.

## Materials and methods

*Aplysia californica* from the University of Miami National Resource for Aplysia reared from egg masses of wild-caught animals were used in these experiments. Animals from each hatchery-reared cohort were from a single egg mass and thus represent either full or half siblings. For selected experiments, wild-caught adults of indeterminate age but that were sexually mature were used. Animals were fed an ad-libitum diet consisting of *Gracilaria ferox* and *Agardhiella subulata* and reared as described previously (Gerdes and Fieber, 2006). Four hatchery-reared animals of different weights were assigned to each cage to allow for identification of individual animals by monthly weight measurements. Sexual maturity for a cohort of animals was designated as the day the first egg mass was laid.

### Behavioral experiments

Animals were placed individually in 48 × 27 × 20 cm translucent plastic cages filled with 13–15°C seawater to a depth of 15 cm and allowed to acclimate for 5 min prior to measurements. A trained experimenter who was unaware of the age of the animals conducted behavioral measures.

#### Righting reflex

Times for the righting and tail-elicited tail withdrawal (TWR) reflexes were recorded monthly from sexual maturity until death in 36 animals from cohort 1. To record the time to right, an individual animal was removed from the bottom of the cage and released on its side from the top of the water column. Contact with the bottom of the cage initiated the timed start of the reflex. The reflex was complete once the animal returned to the upright crawling position and initiated its first crawling step, which necessitated its adherence to the substrate. The reflex was measured 3 times with a rest interval of 5 min between trials, and the times averaged. The placement of an individual animal's data was conserved in the data array, such that differences in righting with age could be tracked in individuals. This added robustness to the ANOVA in analysis of differences in righting with age in the cohort (see Results).

#### TWR

The TWR was measured 5 min after the last righting trial in freely behaving animals following a protocol modified from a previous experiment (Watkins et al., 2010). The animal was placed on its foot in the center of the cage without allowing it to adhere to the substrate, and its resting length was measured, which required lightly holding the animal in position to orient a ruler to its total length. The animal was not held against the substrate during measurement, but rather was hovered just above the substrate. The reflex was initiated by pressing a blunted 18 gauge needle to the tip of the animal's tail for 1 s to depress the tissue against the substrate to a depth that was approximately half the thickness of the tail (i.e., if the tail was 1 cm thick, the needle depression depth was ~0.5 cm), causing tail withdrawal toward the center of the body and signifying T_0_ for the reflex. The retracted total length was then measured with the ruler. At this point the experimenter's hands were removed from the cage. Relaxation of the tail to ~30% of original tail length signified the end of the reflex and T_final_. The reflex was recorded 3 times in each animal, with a rest interval of 10 min between trials. Time and length data from the 3 trials were averaged. Individuals' data in the ANOVA were organized as for the righting reflex.

Wild caught animals maintained in the hatchery for 2–5 days prior to testing were also subjected to tail withdrawal and righting measurements for comparison with hatchery-reared cohorts. The mean values for these behaviors are illustrated as dashed lines in Figures 2B,C.

#### Biting response

The biting response was measured every 2 mos from sexual maturity through senescence in 36 animals from cohort 2 following a protocol modified from previous experiments (Scott et al., 1995; Baxter and Byrne, 2006). Animals were starved for 2 days prior to biting measurements. A paintbrush served as an unconditioned tactile stimulus to initiate biting behavior in unhandled animals presented upright in the rearing cage. The hairs of the paintbrush were positioned to contact the lips for 5 s. This mechanical stimulus to the lips minimized chemosensory cues of the biting response. The amplitude of the biting response was quantified using an arbitrary rating scale of 0–4 (arbitrary units, a.u.s, Scott et al., 1995). In a type 4 response, radular protraction and mouth opening were maximal. In a type 3 response, mouth opening was maximal, but radular protraction was clearly weaker. In a type 2 response, radular protraction was at a minimum but was still visible. In a type 1 response, only slight mouth opening was seen. A type 0 response indicated no biting behavior following stimulation.

The latency of the biting response was measured as the time period from application of the stimulus to maximal radular opening. Measurements of the biting response were recorded 3 times in each animal, with a rest interval of 10 min between trials, and data from the 3 trials were averaged. For analysis, biting data were organized as for the ANOVAS for righting and the TWR.

### Electrophysiological experiments

Whereas cohort 1 was used exclusively for behavioral experiments throughout maturity and senescence, members of cohort 2 and 3 were used for electrophysiological experiments. Before sacrifice animals were subjected to behavioral measurements of the TWR and biting response. Electrophysiological experiments were done at two points in adult life, *mature* (M) and *old* (O). *Mature* animals were age 7–8 mos and sexually reproductive. *Old* animals were age 12–13 mos, corresponding to ages found in cohort 1 to have altered measures of the righting reflex, TWR, and biting response compared to mature.

#### Whole-cell voltage clamp of cultured PVC and BSC sensory neurons

Primary cultures of PVC and BSC neurons were prepared according to previously reported methods (Fieber, 2000; Fieber et al., 2013). Cells were isolated from ganglia, dissociated onto 35 × 10 mm polystyrene culture plates coated with poly-D-lysine, and stored at 17°C in a humidified atmosphere. Whole-cell voltage clamp recordings were made from sensory neurons 24–48 h after plating using 1.5 mm diameter borosilicate glass microelectrodes and an Axopatch 200B patch clamp amplifier connected to a PC equipped with a Digidata 1440 A/D converter and pClamp10 software (Molecular Devices, Sunnyvale, CA). Experiments were performed in continuously flowing artificial seawater (ASW). L-Glu or D-Asp was applied at 1 mM in ASW for 100-ms via a micropipette attached to a picospritzer positioned ~30 μm from the cell body at an angle of ~45° with the perfusion outflow pipette. Applications of agonist were separated by an interval of 60 s to avoid possible desensitization of D-Asp-activated currents or potentiation of L-Glu-activated currents (Carlson and Fieber, 2011) evoked from a holding potential of −70 mV. The order of these agonists was alternated with each cell studied.

#### Intracellular recording in semi-intact and isolated ganglion preparations

For experiments on the electrophysiological changes in aspects of the TWR with aging, a previously described intact tail preparation was used consisting of the left or right pleural-pedal hemiganglia connected to the tail by nerve p9, with all other connectives severed (Clatworthy and Walters, 1993). Ganglia and tail were pinned tightly to a Sylgarded dish. Ganglia were surgically desheathed and maintained in ASW via a gravity-fed perfusion pipette located ~5 mm from the ganglia for the duration of the experiment. Tail PVC sensory neurons in intact ganglia were identified according to electrophysiological criteria detailed previously (Walters et al., 2004). Tail PVC neurons had resting potentials of −35 to −55 mV and were not spontaneously active, but when receptive fields on the tail were stimulated, produced action potentials (APs) of 50–100 mV amplitude. Tail pedal motoneurons P5-7 were identified according to the methods of previous experiments (Walters et al., 1983b). Tail motoneurons were spontaneously active with resting potentials of −40 to −70 mV and were further identified by increased AP firing in response to mechanical stimulation of the tail.

To investigate electrophysiological changes in BSC neurons with aging, an intact buccal ganglion preparation was used. In this preparation all nerve connectives surrounding the buccal ganglion were severed. BSC neurons had resting potentials of −45 to −55 mV, were not spontaneously active, and produced APs in response to intracellular current injection or excitatory agonist application of 85–105 mV amplitude with spike durations ~50–100% longer than PVC neurons.

For intracellular recordings, glass capillary microelectrodes of 5–15 MΩ resistance were used. All recordings were made at room temperature of ~23°C using pClamp10 software with BRAMP-01R and ELC-01MX amplifiers (ALA Scientific Instruments, Farmingdale, NY) connected to a PC and Digidata 1440A A/D converter. L-Glu or D-Asp was applied to neurons *in situ* as a 10 μl bolus at 1 mM in ASW from a manual pipettor (Rainin, Oakand, CA) positioned ~3 mm from the recorded neuron. Input resistance was obtained by measuring the voltage deflection during 0.5 nA, 1 s hyperpolarizing current pulses. AP duration was quantified as the time from AP peak to the return to resting potential (Clatworthy and Walters, 1994). AP duration and amplitude were calculated from the first agonist-evoked AP if multiple spikes were elicited.

To measure electrical excitability in tail PVC and BSC sensory neurons and in tail motoneurons, a protocol was modified from existing procedures (Cleary et al., 1998; Chin et al., 1999). The threshold to fire an AP was measured by applying depolarizing current in increasing increments of 0.1 nA for 500 ms in tail PVC and BSC neurons and for 2 s in tail motoneurons. The threshold to fire was defined as the minimum current required to initiate a single AP. Tail motoneurons were held at −90 mV to arrest free spiking. Excitability was defined as the number of APs evoked in response to a 1.5 nA depolarizing current for 500 ms in tail PVC and BSC neurons and for 2 s in tail motoneurons. Responses in tail motoneurons were also recorded during 70 ms, 1 mA tail shock as a weak electrical stimulus. Tail shock-induced polysynaptic EPSPs were defined as positive voltage deflections greater than twice the amplitude of background noise (e.g., if background noise was 2 mV in amplitude, evoked EPSPs must have been ≥4 mV to be counted). The amplitude of the first evoked EPSP following tail shock was averaged in mature and in old tail motoneurons and compared. The number of EPSPs for up to 5 s following tail shock was averaged in mature and in old tail motoneurons and compared.

#### Solutions

Extracellular solution consisted of ASW containing (mM) 417 NaCl, 10 KCl, 10 CaCl_2_, 55 MgCl_2_, and 15 HEPES-NaOH, pH 7.6. Intracellular solution for whole-cell voltage clamp recordings consisted of (mM) 458 KCl, 2.9 CaCl_2_ (2 H_2_O), 2.5 MgCl_2_ (6 H_2_O), 5 Na_2_ATP, 1 EGTA, and 40 HEPES-KOH, pH 7.4. The pipette solution for intracellular recordings in intact ganglia consisted of 3 M KCl. Solutions containing 1 mM L-Glu or D-Asp were prepared daily from 0.5 M stock solutions by the addition of ASW as previously reported (Carlson et al., 2012). All reagents were from Sigma-Aldrich (St. Louis, MO, USA).

### Data acquisition and analysis

Data are expressed as mean ± SE. For behavioral experiments, significant differences were assessed via one-way within subjects (repeated-measures) ANOVA of mean behaviors, with Tukey's *post-hoc* test. For electrophysiological experiments, significant differences among mean measurements of neuron responses were assessed via 2-sample *t*-test or χ^2^-squared test of independence when appropriate. All analyses were performed using the open source R statistical program (Vienna, Austria). Differences at *p* ≤ 0.05 were accepted as significant. ^*^denotes significance at *p* ≤ 0.05, ^**^at *p* ≤ 0.01, ^***^at *p* ≤ 0.001.

## Results

**Figure 1:**
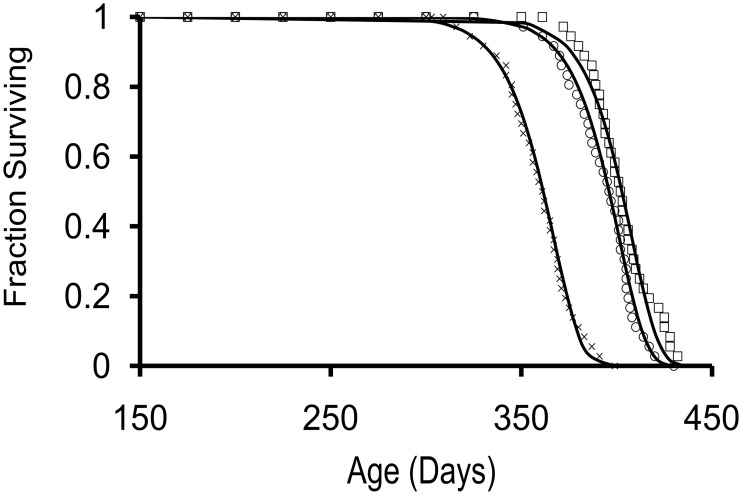
**Survivorship curves for 3 cohorts of hatchery-reared *Aplysia californica* with lifespans of approximately 1 year**. Survivorship curves were fit to a Gompertz survival function, s=exp [(A/G) (1-e^Gt^)], indicated with squares, crosses, and circles for cohort 1, 2, and 3, respectively; starting *n* = 36 for each cohort.

**Figure 2:**
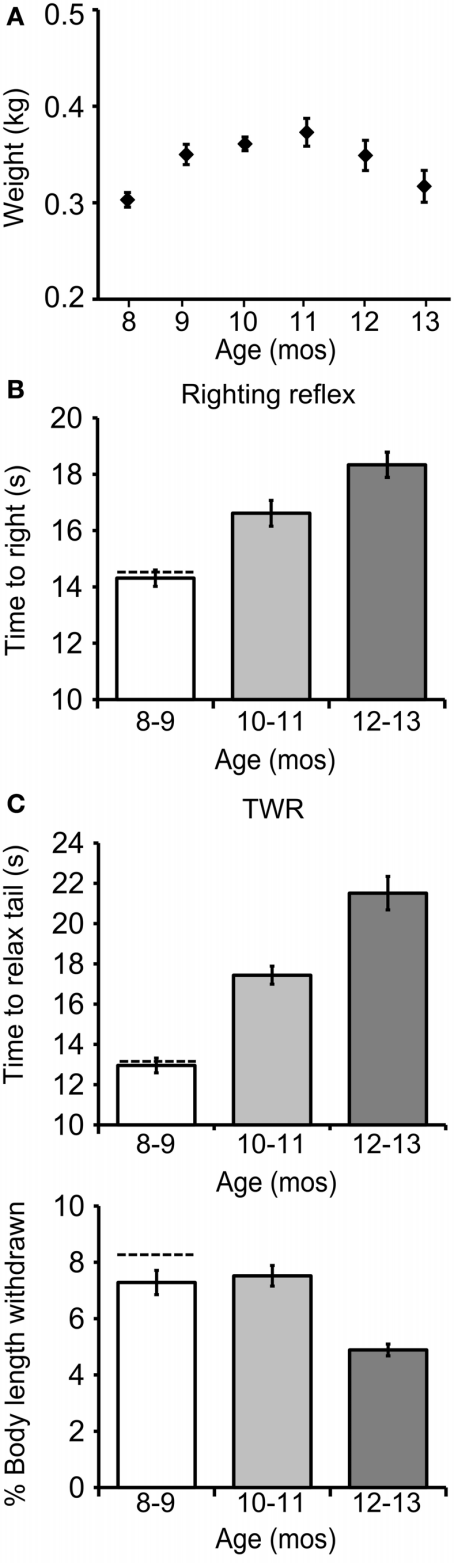
**The righting and tail withdrawal reflexes declined during aging. (A)** Weights for cohort 1 at age 8–12 mos (all data presented as mean ± SE). **(B)** The time to right increased significantly with age [*p* ≤ 0.0001, *F*_(2, 98)_ = 19.1, repeated measures ANOVA]. **(C)** The time to relax tail following tail touch, a measure of the duration of the TWR, increased significantly with age [*p* ≤ 0.0001, *F*_(2, 98)_ = 51.5]. The fraction of starting body length withdrawn following tail touch, a measure of the amplitude of the TWR, declined significantly with age [*p* ≤ 0.0001, *F*_(2, 98)_ = 11.8; *n* = 36].

For the 3 cohorts studied, sexual maturity occurred by 7 mos of age. A subset of 36 animals from each cohort was used to determine survivorship; the data are shown in Figure 1. Median lifespans were 402, 362, and 395 days for cohorts 1–3, respectively. Survivorship curves for each cohort were best fitted by a Gompertz survival function s=exp [(A/G) (1-e^Gt^)] (Wilson, 1994). Calculated aging rates (G) for the 3 cohorts were comparable to previously reported values for hatchery-reared Aplysia (Gerdes and Fieber, 2006) at G ≤ 0.44. As in several other studies from our laboratory (Capo et al., 2003; Gerdes and Fieber, 2006), steady increases in length and weight were observed that peaked during the months immediately following sexual maturity and maximal egg production. A significant reduction in body mass was observed in the weeks immediately preceding death. Body mass decreased significantly from age 11 mos to age 13 mos in cohort 1 (Figure 2A; *p* ≤ 0.05, 2-sample *t*-test).

### The righting reflex and TWR declined during aging

The mean time to right increased significantly with age in cohort 1 [14.3 ± 0.3 s at age 8–9 mos, 16.6 ± 0.5 s at 10–11 mos, and 18.3 ± 0.5 s at 12–13 mos; Figure 2B; *p* ≤ 0.0001, *F*_(2, 98)_ = 19.1, repeated measures ANOVA; Tukey's *post-hoc* analysis, *p* ≤ 0.01 for age 8–9 vs. 10–11 mos, *p* ≤ 0.01 for age 10–11 vs. 12–13 mos, *p* ≤ 0.001 for age 8–9 vs. 12–13 mos]. The time to relax the tail after tail touch, a measure of the duration of the TWR, also increased significantly with age [13.0 ± 0.4 s at 8–9 mos, 17.4 ± 0.5 s at 10–11 mos, and 21.5 ± 0.8 s at 12–13 mos; Figure 2C; *p* = 0.0001, *F*_(2, 98)_ = 51.5; Tukey's *post-hoc* analysis, *p* ≤ 0.01 for age 8–9 vs. 10–11 mos, *p* ≤ 0.01 for age 10–11 vs. 12–13 mos, *p* ≤ 0.001 for age 8–9 vs. 12–13 mos], while the fraction of body length withdrawn, a measure of the amplitude of the TWR, decreased significantly [7.3 ± 0.4% at 8–9 mos, 7.5 ± 0.4% at 10–11 mos, and 4.9 ± 0.2% at 12–13 mos; *p* ≤ 0.0001, *F*_(2, 98)_ = 11.8; Tukey's *post-hoc* analysis, *p* ≤ 0.01 for age 10–11 vs. 12–13 mos, *p* ≤ 0.01 for age 8–9 vs. 12–13 mos].

The righting reflex and TWR were studied in 12 wild-caught animals, acquired by the National Resource for Aplysia as brood stock, and held 2 days prior to testing. Wild-caught animals had a mean time to right of 14.6 ± 1.1 s, a mean tail relaxation time of 13.2 ± 0.5 s, and a mean body withdrawal in TWR of 8.5 ± 0.4% (±SE; dashed horizontal lines in Figures 2B,C). In all these respects wild animals were comparable to hatchery-reared animals at ages 8–9 mos.

### The biting response declined during aging

**Figure 3:**
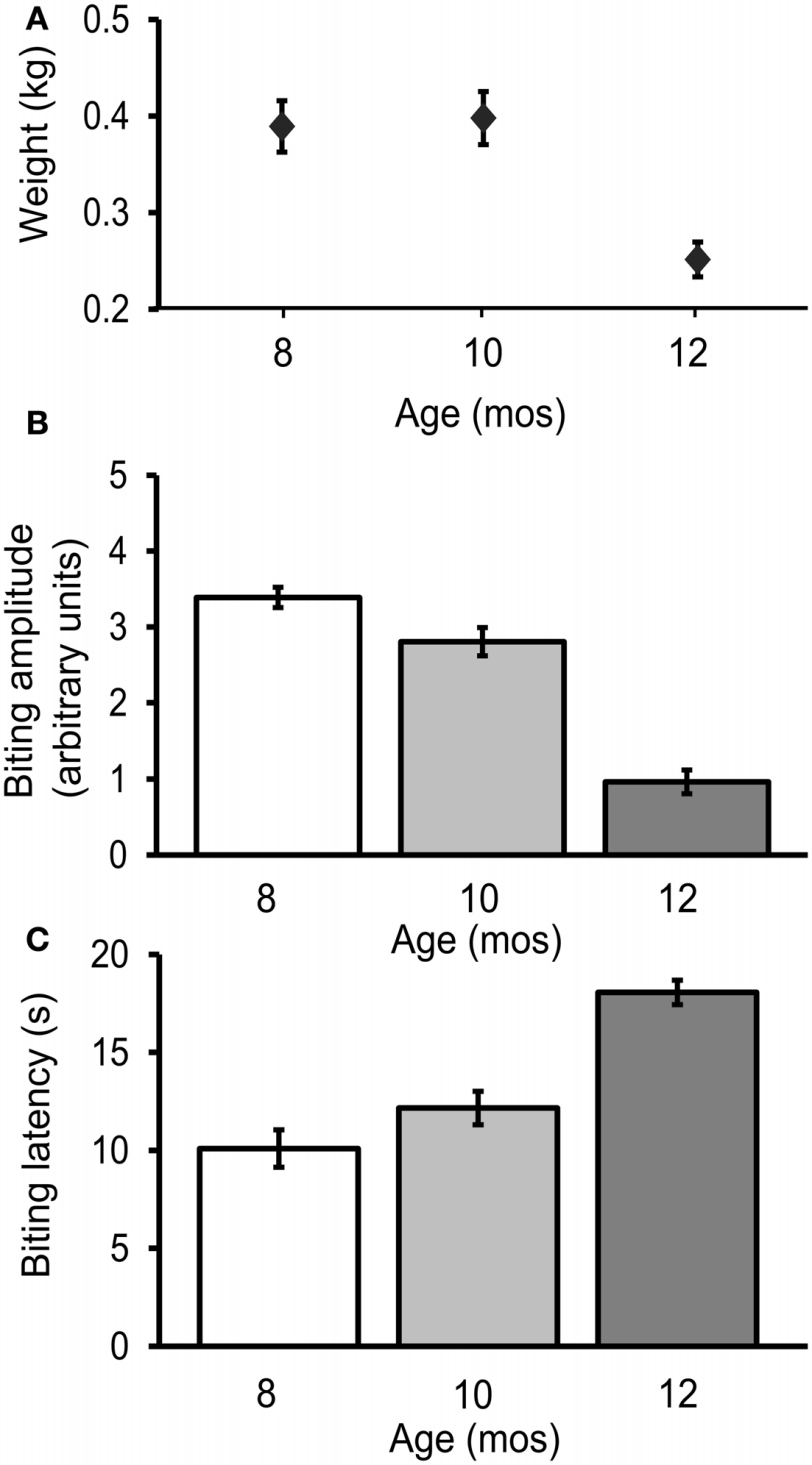
**The biting response declined during aging. (A)** Weight decreased significantly with age [*p* ≤ 0.01, *F*_(2, 98)_ = 7.1, repeated measures ANOVA]. **(B)** Biting amplitude decreased significantly with age [*p* = 0.001, *F*_(2, 98)_ = 9.3] **(C)** The latency between stimulus contact to the lips and first bite increased significantly with age [*p* = 0.0001, *F*_(2, 98)_ = 21.2; *n* = 36].

The biting response was measured every 8 weeks during adult life in 36 freely behaving hatchery reared cohort 2 animals. Biting amplitude decreased significantly with age [3.4 ± 0.1 arbitrary units, a.u.s, at age 8 mos, 2.8 ± 0.2 a.u.s at 10 mos, and 1.0 ± 0.2 a.u.s at 12 mos; Figure 3B; *p* ≤ 0.001, *F*_(2, 98)_ = 9.3; Tukey's *post-hoc* analysis, *p* ≤ 0.05 for age 8 vs. 10 mos, *p* ≤ 0.01 for age 10 vs. 12 mos, *p* ≤ 0.001 for age 8 vs. 12 mos]. The latency between probe contact with the lips and the first bite increased significantly with age [10.1 ± 1.0 s at age 8 mos, 12.2 ± 0.9 s at 10 mos, and 18.1 ± 0.6 s at 12 mos; Figure 3C; *p* ≤ 0.0001, *F*_(2, 98)_ = 21.2; Tukey's *post-hoc* analysis, *p* ≤ 0.05 for age 8 vs. 10 mos, *p* ≤ 0.01 for age 10 vs. 12 mos, *p* ≤ 0.01 for age 8 vs. 12 mos]. Coincidentally, mean weight decreased significantly with age [Figure 3A; *p* ≤ 0.01, *F*_(2, 98)_ = 7.1; Tukey's *post-hoc* analysis, *p* ≤ 0.01 for age 10 vs. 12 mos, *p* ≤ 0.01 for age 8 vs. 12 mos].

### Behavioral aging was associated with decreased glutamatergic responses in SNs

**Figure 4:**
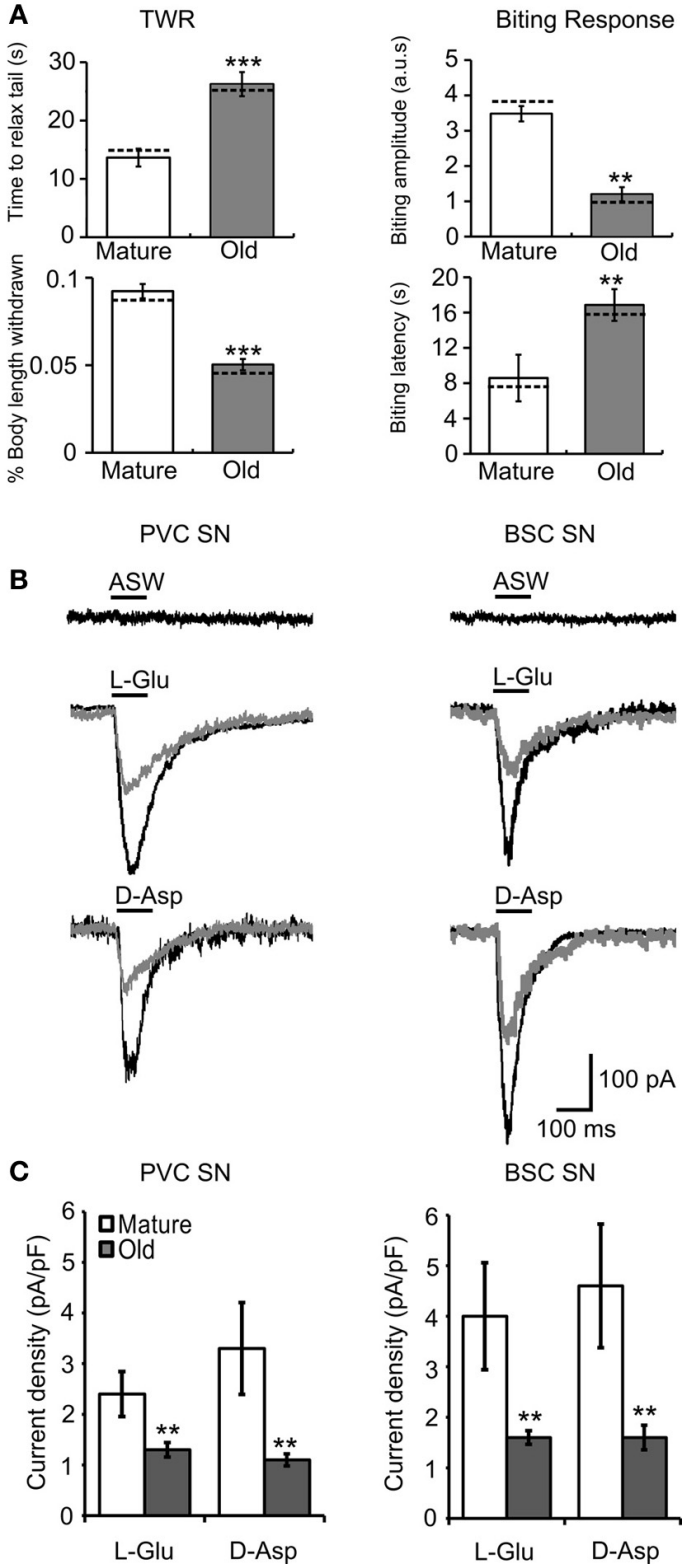
**Behavioral declines during aging correspond to altered agonist-induced responses in cultured PVC and BSC sensory neurons. (A)** Behavioral measures in animals from cohort 2 sacrificed for electrophysiological experiments. The time to relax tail following tail touch increased and fraction of body length withdrawn decreased significantly with age (*p* ≤ 0.001 in each case, 2-sample *t*-tests). The biting latency increased and biting amplitude decreased significantly with age (*p* ≤ 0.01 in each case; *n* = 14 for M, *n* = 12 for O). Dotted horizontal lines are individual behavioral measurements in animals whose electrophysiological responses are illustrated in **(B)**. **(B)** Representative whole cell currents evoked in response to a 100 ms pulse of 1 mM L-Glu in mature and old PVC and BSC neurons. No current was evoked in response to a 100 ms pulse of ASW alone. **(C)** Excitatory currents evoked by L-Glu and D-Asp decreased significantly with age in PVC (*n* = 14 for M, *n* = 23 for O) and BSC neurons (*n* = 22 for M, *n* = 15 for O; *p* ≤ 0.01 in each case, 2-sample *t*-tests). ^**^ and ^***^ denote significant difference from mature at *p* ≤ 0.01 and *p* ≤ 0.001, respectively.

Aging-related changes in the neuronal circuitry corresponding to these behaviors were investigated. Cohorts 2 and 3 were used in electrophysiological experiments at ages 7–8 (M) and 12–13 mos (O), consistent with the ages at which behavioral changes were observed (Figures 2, 3). The behavioral data for animals used in electrophysiology are summarized in Figures 4A, 6A. The means were within the ranges reported above, moreover, similar significant aging differences were noted. Dashed horizontal bars are included that represent the individual behavioral measurements of animals whose electrophysiological data is presented in the figures. Mean resting membrane potential, input resistance, AP duration, and AP amplitude did not change with aging in any neuronal type investigated (Table 1), but were consistent with previously published values for healthy tail PVC and BSC neurons and tail motoneurons (Zhang et al., 1994; Walters et al., 2004; Fieber et al., 2010).

**Table 1:** **Resting membrane potential, input resistance, spike duration, and spike amplitude were not significantly different in intact neuronal preparations during aging**.

	**Tail PVC**		**Tail MN**		**BSC**	
	**Mature**	**Old**	**Mature**	**Old**	**Mature**	**Old**
Resting membrane potential (mV)	−45.2 ± 5.8	−44.4 ± 65.9	−52.1 ± 4.9	−53.9 ± 9.8	−44.8 ± 5.3	−43.2 ± 3.0
Input resistance (MΩ)	25.4 ± 4.2	24.9 ± 4.8	10.1 ± 1.9	9.4 ± 2.4	18.2 ± 3.9	16.4 ± 5.1
Spike duration (ms)	3.6 ± 0.5	3.9 ± 1.0	4.3 ± 0.8	4.4 ± 0.7	6.2 ± 0.7	6.0 ± 1.4
Spike amplitude (mV)	85.3 ± 15.1	75.7 ± 14.7	72.4 ± 18.2	78.9 ± 13.3	97.4 ± 4.8	96.0 ± 7.4

Picospritzer pulse-application of L-Glu or D-Asp evoked an inward current in cultured mature and old sensory neurons voltage clamped at −70 mV, presumably via activation of iGluR, while application of ASW alone did not induce currents (Figure 4B). Mean L-Glu current density (*M* = 2.4 ± 0.5 pA/pF, *O* = 1.3 ± 0.2 pA/pF) and D-Asp current density (*M* = 3.4 ± 0.9 pA/pF, *O* = 1.2 ± 0.1 pA/pF) decreased with age in PVC neurons (*p* ≤ 0.01 in each case, 2-sample *t*-tests; Figures 4B,C, left). In BSC neurons, mean L-Glu current density (*M* = 4.0 ± 0.9 pA/pF, *O* = 1.7 ± 0.2 pA/pF) and D-Asp current density (*M* = 4.6 ± 1.1 pA/pF, *O* = 1.6 ± 0.3 pA/pF) also decreased with age (*p* ≤ 0.01 in each case; Figures 4B,C, right). Fewer old PVC neurons responded to L-Glu (*M* = 52%, *O* = 38%) or D-Asp (*M* = 39%, *O* = 26%; *p* ≤ 0.01 in each case, χ^2^ tests of independence). The same aging deficits were observed in BSC neurons (L-Glu responses: *M* = 69%, *O* = 43%, D-Asp responses: *M* = 81%, *O* = 57%, *p* ≤ 0.01 in each case).

**Figure 5:**
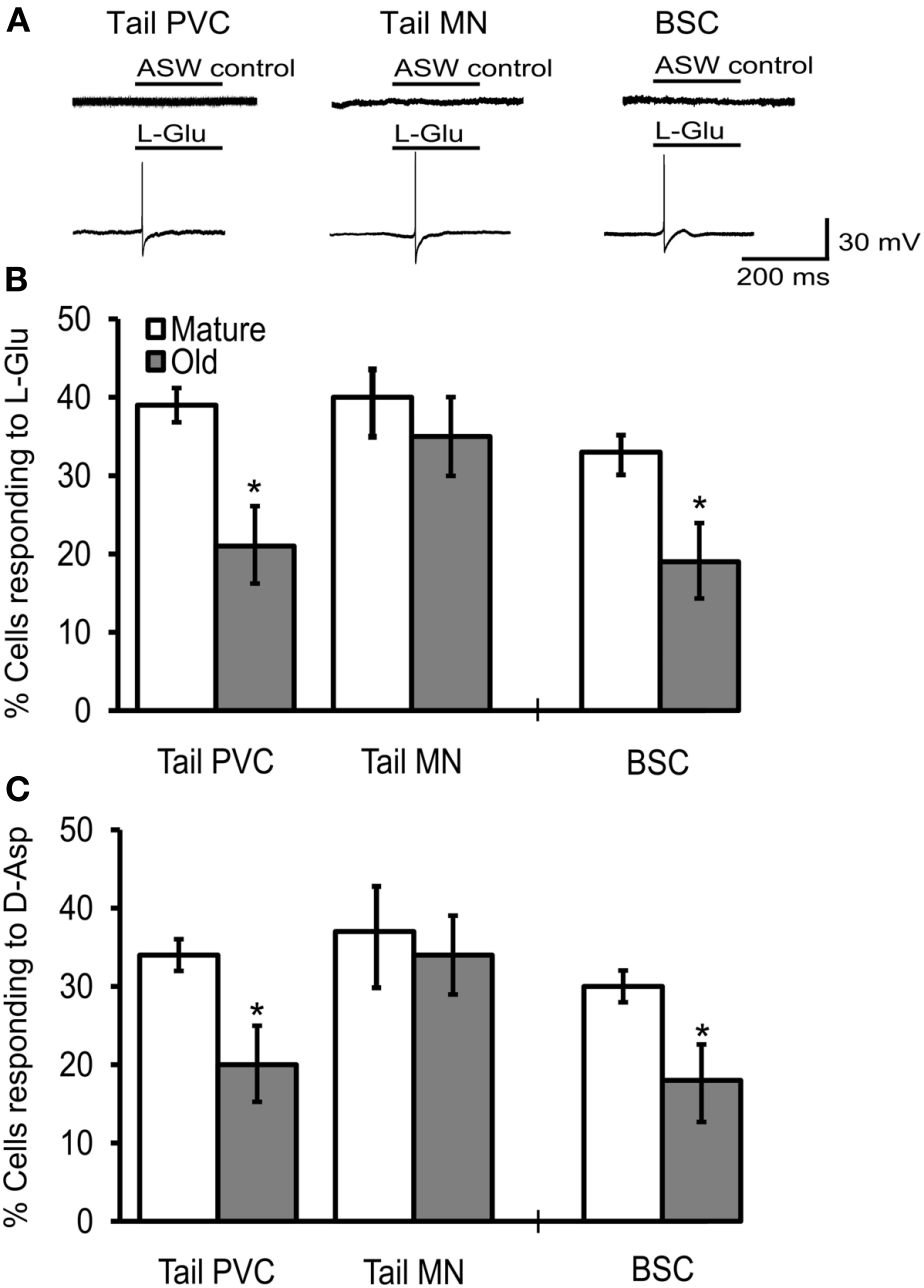
**The frequency of cells responding to excitatory agonists with an AP declined in tail PVC and BSC sensory neurons of intact preparations. (A)** Application of 1 mM L-Glu or D-Asp evoked excitatory discharge in sensory and motor neurons of intact preparations while application of ASW alone did not. Tail PVC and BSC sensory neurons were quiescent until agonist was applied. Tail motoneurons were held at −90 mV to arrest free spiking. **(B)** The frequency of PVC and BSC sensory neurons responding to L-Glu with APs decreased significantly with age (*p* ≤ 0.05 in each case, 2-sample *t*-tests) while in tail motoneurons no change was observed. **(C)** The frequency of PVC and BSC sensory neurons responding to D-Asp with an AP also decreased significantly with age (*p* ≤ 0.05 in each case) while in tail motoneurons no change was observed (Tail PVC: *n* = 41 for M, *n* = 42 for O; Tail MN: *n* = 27 for M, *n* = 25 for O; BSC: *n* = 14 for M, *n* = 15 for O). ^*^ denotes significant difference from mature at *p* ≤ 0.05.

In reduced preparations, application of 1 mM L-Glu evoked AP firing in tail PVC neurons, tail motoneurons, and BSC neurons (Figure 5A). The frequency of PVC neurons firing an AP from rest in response to L-Glu application (*M* = 39%, *O* = 21%; Figure 5B) or D-Asp application (*M* = 34%, *O* = 20%; Figure 5C) decreased significantly with age (*p* = 0.05 in each case, χ^2^ tests of independence). Similarly, the frequency of BSC neurons with L-Glu responses (*M* = 33%, *O* = 19%; Figure 5B) or D-Asp responses (*M* = 30%, *O* = 18%; Figure 5C) decreased significantly with age (*p* = 0.05). Like the sensory neurons studied here, pedal motoneurons are glutamatergic (Chitwood et al., 2001). In P7-9 pedal motoneurons innervating the tail, the frequency of L-Glu responses (*M* = 40%, *O* = 35%; Figure 5B) and D-Asp responses (*M* = 37%, *O* = 34%; Figure 5C) were unaffected with age.

### Behavioral aging was associated with altered responses to depolarizing current injection in SNs

**Figure 6:**
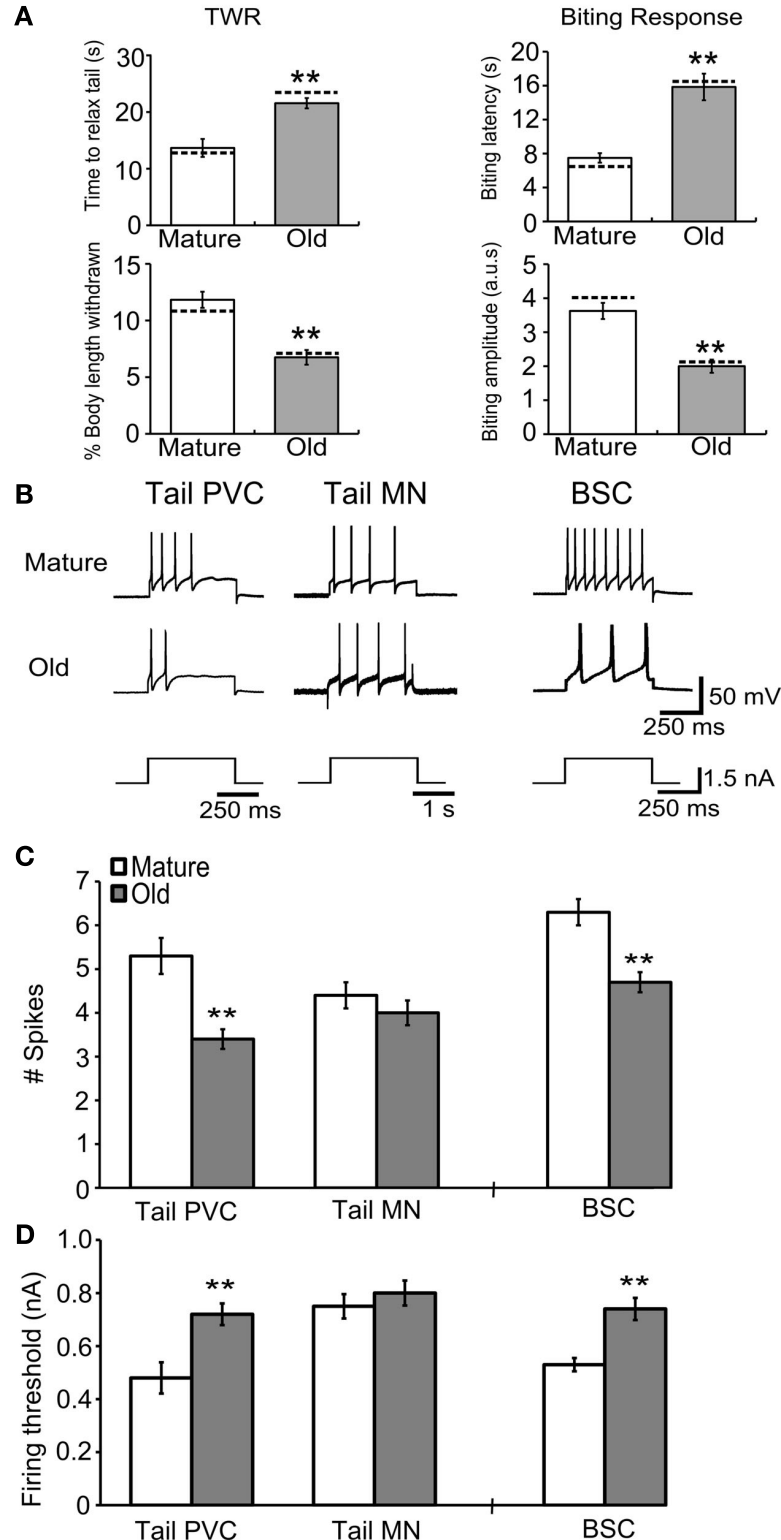
**Behavioral declines during aging correspond to changes in response to current injection in tail and buccal sensory neurons. (A)** The time to relax tail following tail touch increased and percent of body length withdrawn decreased significantly with age in cohort 3 (*p* ≤ 0.01 in each case, 2-sample *t*-tests). The biting latency increased and biting amplitude decreased significantly with age (*p* ≤ 0.01 in each case; *n* = 8 for M, *n* = 9 for O). Dotted horizontal lines are individual behavioral measurements in animals used for electrophysiology in **(B)**. **(B)** Representative responses during 1.5 nA current injection for 500 ms in tail PVC and BSC sensory neurons and 2 s in tail motoneurons. **(C)** The number of APs fired during 1.5 nA current injection decreased significantly with age in tail PVC and BSC sensory neurons (*p* ≤ 0.01 in each case) while in tail motoneurons no change was observed. **(D)** Firing threshold increased significantly with age in PVC and BSC sensory neurons (*p* ≤ 0.01 in each case) while in tail motoneurons no change was observed (Tail PVC: *n* = 26 for M, *n* = 24 for O; Tail MN: *n* = 19 for M, *n* = 18 for O; BSC: *n* = 16 for M, *n* = 23 for O). ^**^ denotes significant difference from mature at *p* ≤ 0.01.

Intact neurons were subjected to depolarizing intracellular current pulses to measure changes in their electrical excitability with aging. The mean number of APs fired during 500 ms, 1.5 nA current injection decreased significantly in PVC (*M* = 5.3 ± 0.4 spikes, *O* = 3.4 ± 0.2 spikes) and BSC sensory neurons (*M* = 6.3 ± 0.3 spikes, *O* = 4.7 ± 0.2 spikes; Figures 6B,C; *p* ≤ 0.01 in each case, 2-sample *t*-tests), while in tail motoneurons 1.5 nA, 2 s current injection produced no change in spike number during aging (*M* = 4.4 ± 0.3 spikes, *O* = 4.0 ± 0.3 spikes). The mean threshold for firing an AP increased significantly with age in PVC (*M* = 0.48 ± 0.06 nA, *O* = 0.72 ± 0.04 nA) and BSC neurons (*M* = 0.53 ± 0.03 nA, *O* = 0.74 ± 0.04 nA; Figure 6D; *p* ≤ 0.01 in each case, 2-sample *t*-tests), while no change was observed in tail motoneurons during aging (*M* = 0.75 ± 0.05 nA, *O* = 0.80 ± 0.05 nA).

### Responses of tail MNs to tail shock changed during aging

**Figure 7:**
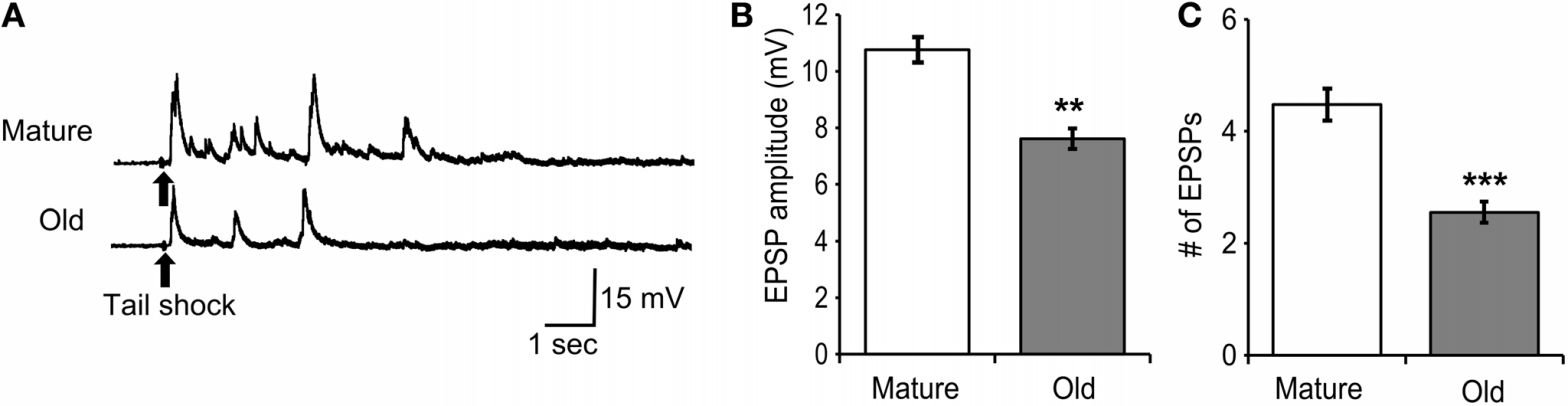
**Responses of tail motoneurons *in situ* following 70 ms, 1 mA tail shock declined with age. (A)** Representative responses in mature and old tail motoneurons following tail shock. Motoneurons were hyperpolarized to −90 mV. **(B)** The amplitude of the first evoked EPSP decreased significantly with age (*p* ≤ 0.01; 2-sample *t*-test). **(C)** The number of EPSPs evoked for up to 5 s following tail shock also decreased significantly with age (*p* ≤ 0.001; *n* = 21 for M, *n* = 27 for O). ^**^ and ^***^ denote significant difference from mature at *p* ≤ 0.01 and *p* ≤ 0.001, respectively.

Tail motoneurons were monitored at a holding potential of −90 mV during 70 ms, 1 mA tail shock (Figure 7A). The amplitude of the first evoked EPSP was averaged between cells and found to decrease significantly with age (*M* = 10.8 ± 0.5 mV, *O* = 7.6 ± 0.4 mV; Figure 7B; *p* ≤ 0.01, 2-sample *t*-test). The number of EPSPs evoked for up to 5 s following tail shock was averaged between cells and found to decrease significantly with age (*M* = 4.5 ± 0.3, *O* = 2.6 ± 0.2; Figure 7C; *p* ≤ 0.01, 2-sample *t*-test).

## Discussion

Aging research has identified specific changes in physiological pathways attributed to behavioral declines in several model species. However, it is difficult to attribute these changes to defined neuronal circuits in most animal models. Using the simple Aplysia neurophysiological model, this study found changes in behavioral measures that were attributed in part to altered nervous system functioning. Specifically, we found that aging changes in the TWR and biting response were linked to declines in sensory neuron excitability.

While some biological markers of aging have been identified in Aplysia including shell size, weight, and changes in hemolymph protein levels (Peretz and Srivatsan, 1992), improved markers are both practically useful and would more precisely define aging in this model organism. One of the goals of this study was to establish stages of aging in Aplysia based on morphology and quantitation of non-invasive behaviors. In every cohort studied, behavioral assessments of aspects of the righting reflex, TWR, and biting response changed in a consistent direction with age. According to the developmental stages that are widely used in the Aplysia community (Kriegstein, 1977), the last stage recognized is mature stage 13, marked by the formation of the genital groove and the genital ganglion, and egg laying. Stage 13 encompasses an animal mass range of g-kg. Defining additional stages in the adult life cycle would provide reference points for understanding variations in certain electrophysiological responses observed and otherwise attributed to the use of non-inbred animals.

Adult Aplysia may be divided into 3 stages of aging based on significant differences in behavioral and morphological indices found in this study: *Mature, Aged I, and Aged II* (Table 2). *Mature* animals correspond to age 7–9 mos in cohorts 1, 2, and 3. *Mature* animals are within 90 d after first egg laying, and near peak mass and egg production. Their behavioral times are righting ≤15.0 s, recovery from tail touch ≤13.0 s, biting amplitude ≥3.0 a.u.s, and biting latency ≤11.0 s. They correspond to the time point *mature* (M) in electrophysiological experiments. *Aged I* animals correspond to age 10–11 mos and are 91–120 d after first sexual maturity, but already demonstrate significant declines in behavioral responses such as righting 15.1–18.0 s, recovery from tail touch 13.1–20.0 s, biting amplitude 2.0 a.u.s, and biting latency 11.1–15.0 s. *Aged II* animals correspond to > age 11 mos and are sexually mature ≥121 d. They correspond to the time point *old* (O) in electrophysiological experiments. *Aged II* animals have significantly decreased mass compared to *Aged I*, with righting ≥18.1 s, recovery from tail touch ≥20.1 s, biting amplitude ≤1.0 a.u.s, and biting latency ≥15.1 s. Thus, animals reaching sexual maturity at age 7 mos would be *Mature* from 7 to ≤10 mos, *Aged I* from 10 to ≤11 mos, and *Aged II* from age >11 mos using these criteria. Although the behavioral measures for *Mature, Aged I*, and *Aged II* would require a practiced experimenter to apply to animals of unknown age with any reliability, they allow improved estimates of age in wild animals of varying mass prior to electrophysiological assessments common to the Aplysia neurobiology community, including aging or learning studies. As age-related changes in behavior were found in this study and others (Rattan and Peretz, 1981; Peretz and Srivatsan, 1992), stage of life assessments are crucial for understanding data generated from experiments involving behavior.

**Table 2:** **Stages of aging in Aplysia based on age and behavior**.

**Stage of aging**	Age (mos)^*^	**Days after 1st eggs**	**Righting (s)**	**Time to relax tail (s)**	**Biting amp (a.u.s)**	**Biting latency (s)**
Mature	7–9	≤90	≤15.0	≤13.0	3.0	≤11.0
Aged I	10–11	91–120	15.1–18.0	13.1–20.0	2.0	11.1–15.0
Aged II	>11	>121	≥18.1	≥20.1	≤1.0	≥15.1

* *based on 12 mo lifespan*.

A second goal of this study was to determine whether age-related changes in tail withdrawal and biting were associated with changes in relevant sensory or motor neuron function in *in situ* preparations. We also wondered if the sensory and motor neural circuitry aged concurrently or differentially. This study provided evidence for a relationship between behavioral aging and declines in excitability in sensory neurons conveying tactile stimuli that are associated with the behaviors. Our results indicated that specific simple behaviors in Aplysia aged, and some of the neural components of such behaviors also appeared to change while others did not. Specifically, sensory neuron physiology showed aging declines but motor circuitry of the TWR did not, supporting the hypothesis that different parts of the nervous system age differently (Moroz and Kohn, 2010). Thus, behavioral aging is connected to electrophysiological proxies of aging in sensory neurons that serve the behaviors.

Previously, responses of isolated PVC and BSC neurons to D-Asp were shown to decline with aging (Fieber et al., 2010). We confirmed the decrease in D-Asp-evoked responses during aging in cultured PVC and BSC neurons and extended it to L-Glu-evoked responses. We then saw evidence of the same decreases in excitability with aging in PVC and BSC neurons of intact ganglia whether we measured excitatory agonist-evoked or depolarizing current-evoked responses. The frequency of responders to excitatory agonist application decreased in old tail PVC and BSC sensory neurons in intact ganglia and isolated neuronal preparations compared to mature. Furthermore, the number of APs evoked by depolarizing current injection decreased while the threshold for firing an AP increased during aging in intact tail PVC and BSC neurons.

Meanwhile, in intact tail motoneurons within pedal ganglia, the fraction of neurons responding to agonist did not change during aging. In addition, no changes in electrical excitability were found in the number of APs elicited during intracellular current injection or the threshold to fire an AP, suggesting that tail motoneurons age at a different rate than tail sensory neurons.

We then studied the consequences of decreased excitability in old PVC neurons on the motoneurons they control, and found a decrease in the average amplitude of the first evoked EPSP following tail shock. PVC neurons have been shown to make strong connections to tail motor neurons in the pedal ganglia (Walters et al., 1983a) and it is likely that the first EPSP evoked following tail shock was the result of PVC input. Thus, the decrease in EPSP amplitude following tail shock in old tail MN may be the result of reduced PVC sensory neuron excitability during aging. Other polysynaptic inputs, such as connections from other sensory neurons and interneurons, contribute to the multiple EPSPs observed for several seconds following tail shock (White et al., 1993). The decrease in old tail MNs in the number of EPSPs evoked for up to 5 s following tail shock may be attributed to a decrease in the number or strength of these polysynaptic connections, which also reflects aging of this circuit.

Previous age-related changes in the input and output properties of motoneurons have been found in Aplysia. In L7 motoneurons, spiking following siphon stimulation was shown to be depressed and PSP amplitude was shown to be reduced in old animals compared to mature (Peretz and Srivatsan, 1992). The ability of L7 to elicit gill contraction was reduced in old animals (Peretz et al., 1982). Although sensory neuron function was not directly studied, observed deficits in motoneuron input and output properties with aging may result from declines in sensory neuron excitability as demonstrated here.

The physiological role of L-Glu and D-Asp excitatory responses in PVC and BSC sensory neurons is undemonstrated. Sensorimotor synapses of these cells are vulnerable to synaptic facilitation and depression (Walters et al., 1983b; Baxter and Byrne, 2006), thus excitatory agonists including L-Glu and D-Asp may serve as neuromodulators. iGluR subunits have been localized to nerve terminals in peripheral tissues served by sensory afferents (Carlton et al., 1995). Administration of L-Glu to peripheral sensory axons resulted in nociceptive behaviors including a significant elevation of rat paw withdrawal scores following mechanical stimulation (Carlton et al., 1995, 1998). L-GluR agonists may stimulate sensory PVC neurons to serve a similar nociceptive role in the Aplysia nervous system.

It has been demonstrated that L-Glu activates an excitatory current in Aplysia PVC and BSC neurons and, based on pharmacological criteria, may activate NMDA-like receptors containing NR2C/D-like subunits (Carlson et al., 2012). Certain classes of iGluRs and their activity have been shown to decline during aging in vertebrates (Segovia et al., 2001; Foster, 2012). It is possible that decreases in iGluR signaling presented here are due to NMDAR dysfunction similar to that seen in vertebrates during aging (Foster et al., 2001). Decreased responses to excitatory agonists during aging have been demonstrated in other neuronal types of the Aplysia nervous system as well. In the bursting neuron R15 of the abdominal ganglia, the bursting pattern following acetylcholine (ACh) application changed with aging while the resting membrane potential and latency in response to ACh did not (Akhmedov et al., 2013).

The consistent decreases in excitatory responses in PVC and BSC sensory neurons and consistent deficits in corresponding behaviors in old Aplysia suggests that behavioral aging correlates with changes in nervous system function. Although atrophy of innervating muscles may underlie changes in behavioral output during aging and were not studied here, the reproducible decreases in excitatory responses in both cultured and intact sensory neurons provide evidence that at least some parts of the neural circuitry also aged. It is likely that behavioral deficits in aged animals are due to both muscle atrophy and nervous system dysfunction.

Our findings demonstrate that behavioral changes may be used in combination with morphological signposts to classify distinct stages in the adult life of Aplysia. Old sensory neurons innervating tail withdrawal and biting behaviors had decreased responses to excitatory agonist application and electrical stimulation while old tail pedal motoneurons did not, suggesting that sensory neuron aging occurs before motoneuron aging in some behaviors. Its compact lifespan as well as these results extend the usefulness of the Aplysia model for linking behavioral aging with corresponding changes in nervous system functioning.

### Conflict of interest statement

The authors declare that the research was conducted in the absence of any commercial or financial relationships that could be construed as a potential conflict of interest.
